# *SMN2* Copy Number Association with Spinal Muscular Atrophy Severity: Insights from Colombian Patients

**DOI:** 10.3390/jcm13216402

**Published:** 2024-10-25

**Authors:** José Lamadrid-González, Sandra Castellar-Leones, Julio César Contreras-Velásquez, Valmore Bermúdez

**Affiliations:** 1 Programa de Maestría en Genética, Centro de Investigaciones en Ciencias de la Vida, Universidad Simón Bolívar, Barranquilla, Atlántico 080003, Colombia; 2 Departamento de Medicina Fisica y Rehabilitacion, Facultad de medicina, Universidad Nacional de Colombia, Bogotá DC 111321, Colombia; smcastellarl@unal.edu.co; 3 Departamento de Productividad e Innovación, Universidad de la Costa, Barranquilla, Atlántico 080002, Colombia; jcontrer30@cuc.edu.co; 4 Facultad de Ciencias de la Salud, Centro de Investigaciones en Ciencias de la Vida, Universidad Simón Bolívar, Barranquilla, Atlántico 080003, Colombia; valmore.bermudez@unisimon.edu.co

**Keywords:** spinal muscular atrophy, *SMN2* copies, phenotype, phenotype variation

## Abstract

**Background:** Spinal muscular atrophy (SMA) is a genetic neurodegenerative disease primarily affecting paediatric patients, often leading to significant morbidity and mortality. Our principal objective is to describe the sociodemographic characteristics and evaluate the association between the number of *SMN2* copies and SMA type in patients from the Colombian Foundation for Spinal Muscular Atrophy (FAMECOL) database. **Methodology:** An analytical cross-sectional study was conducted on 201 patients with a genetic diagnosis of SMA. Data were identified, extracted, and collected from patient records provided by FAMECOL as patients registered with the association, including 201 patients from April 2013 to April 2024, when the database was delivered. Qualitative variables were described using relative and absolute frequencies, while quantitative variables were described using central tendency and dispersion measures according to their distribution. The association between the SMA type and the *SMN2* number of copies was assessed by Fisher’s exact test (1 to 5 copies). **Results:** Of the 201 patients studied, 42% were female (n = 85), and 58% were male (n = 116). The median age was 9 years (IQR 4–16 years). The median age at diagnosis was 9 years (IQR 4–16), varying by subgroup: 2, 7, 14, and 41.5 years for each type, respectively. A total of 25% patients were from Antioquia (n = 51). Eighty-nine per cent had gastrostomy (n = 18). The association between the two variables was statistically significant (*p* < 0.05). **Conclusion:** This study highlights SMA clinical variability and its association with the number of SMN2 copies, underscoring the importance of a personalised approach to diagnosing and managing this disease. The findings may guide more effective therapeutic strategies to improve patients’ quality of life.

## 1. Introduction

Spinal muscular atrophy (SMA) is an autosomal recessive inherited disorder with a variable phenotype, characterised by progressive muscle weakness and reduced muscle tone due to the associated destruction of alpha motor units [1]. SMA was first described by Guido Werdnig in 1891 in a paediatric patient with hypotonia, weakness, and fasciculations [2]. Shortly after, Johann Hoffmann coined the term ‘Spinal Muscular Atrophy’ to use for children from the same family who exhibited identical clinical features, and he described seven cases between 1893 and 1900 [3]. Since then, it has been known as Type 1 spinal muscular atrophy or Werdnig–Hoffmann disease [1,4]. Similarly, Dubowitz depicted a less severe form of SMA in the 1950s, characterised by preserved standing and walking abilities with prolonged survival [5]. Later, its clinical features were described in depth by Eric Kugelberg and Lisa Welander in a research paper published in 1956, so the condition is sometimes called Wohlfart–Kugelberg–Welander disease [6].

This initial work highlighted the anterior spinal horn cell degeneration causing the characteristic proximal symmetric weakness in limb, axial, intercostal, and bulbar muscles as the underlying pathologic and clinical features. Nevertheless, it was not until a century later, with the advent of molecular techniques, that the causative gene and its common variants were identified [7], contributing to the understanding of The International Spinal Muscular Atrophy Consortium SMA classification of 1991 [1], ushering in a new research era focused on developing disease-modifying therapies, including gene therapy [8]. In this regard, SMA is caused by mutations in the survival motor neuron 1 gene (*SMN1*), which is involved in motor neuron functioning via assembly regulation of small nuclear ribonucleoprotein (snRNP) complexes [9].

In addition, humans have an *SMN1* paralog called *SMN2* caused by 5q13 intrachromosomal duplication. The centromeric *SMN2* critically differs from the telomeric *SMN1* at the base pair position 840, a C-to-T substitution that excludes exon 7 from around 90% of the *SMN2* mRNA transcripts [10]. These transcripts lacking exon 7 produce very low SMN protein levels because of their instability and fast degradation [1]. In this regard, a homozygous deletion in the 5q13 region, which encodes the survival motor neuron gene (*SMN1*), causes 95% of SMA cases [11], and the remaining 5% are attributed to pathogenic point mutations [11,12].

The SMA heterogeneity lies in the two forms of the SMN gene—the telomeric variant (*SMN1*) and the centromeric version (*SMN2*)—with individuals varying in the number of *SMN2* copies they possess [13]. *SMN1* transcription produces a fully functional mRNA to perform the SMN protein synthesis [14]. Transcription of *SMN2* results in a fully functional mRNA 10–15% of the time, resulting in much less SMN protein being encoded than *SMN1* [15]. *SMN2* is identical to *SMN1* except for a single CT substitution in exon 7 [16,17]. This substitution promotes splicing 80–85% of the time during transcription and the resulting exclusion of exon 7 [18]. This truncated mRNA results in non-functional proteins. SMA patients lack *SMN1* and rely on *SMN2*, which is residual SMN protein functional by the alpha motor neuron activity and subsequent survival [19]. Thus, a positive correlation is observed between the number of *SMN2* copies and the severity of the phenotype: type 1 SMA patients typically have 1–2 copies of *SMN2*, and type 4 SMA patients have 3–5 copies of *SMN2* [19,20], as shown in Table 1. While the number of SMN2 copies significantly predicts SMA severity, a perfect correlation does not exist due to phenotypic *SMN2* gene variability [21], leading to unpredictable SMN protein production between the affected individuals [22]. Consequently, although cases of a low number of *SMN2* copies with a milder clinical phenotype have been reported, the number of *SMN2* copies remains a consistent factor in predicting the aggressiveness and rate of progression of SMA [22].

SMA is the second cause of death related to autosomal recessive inheritance [7], with an incidence of 1 in 6000 to 11,000 and a carrier frequency of *SMN1* mutations of 2–3% (1 in 40) in the general population [7,8]. SMA incidence varies by ethnicity, being more frequent in Caucasians compared to Blacks or mixed ethnicities [25,26]. For example, the African-descendant population of South Africa present 1 in 3575 live births [27], while in Europeans, it is estimated to be 1 in 3900 to 16,000 live births [8]. On the other hand, there are no estimates of its prevalence in Latin America [12], with no reports in Colombia except for isolated case reports and 42 SMA patients reported at a reference centre in Bogotá [28,29,30]. In this sense, in Colombia and other Latin American countries, there is an assumed problem of underdiagnosis and, consequently, under-reporting [12], as there are no data available to accurately know the prevalence and incidence of this type of disease due to a lack of diagnostic suspicion and untimely diagnosis [31,32].

In 2010, Colombia passed a law that protects patients with orphan diseases, including those with SMA [33]. Currently, two disease-modifying drugs have been approved by INVIMA (the National Institute for Food and Drug Surveillance, Colombia’s regulatory authority for medicines): Nusinersen since 2019 [34] and Risdiplam since 2023 [35]. These drugs have demonstrated through evidence that they alter the natural course of the disease, reducing mortality in patients with SMA type 1 and achieving motor milestones that would not be expected based on the natural phenotype of the disease in treated patients [36]. Therefore, understanding the population characteristics of SMA patients in Colombia allows for better prognosis by knowing the number of *SMN2* copies, population behaviour, and the distribution of the disease, as well as strategies for early diagnosis in areas with the highest number of identified patients.

Therefore, the primary objective of this study is to characterise the largest group of spinal muscular atrophy patients in Colombia, exploring their sociodemographic and genetic features. Additionally, we aim to investigate the possible association between SMA type and the number of *SMN2* gene copies to understand better the Colombian patient population for clinical, prognostic, and public health decision-making regarding this group of patients.

## 2. Materials and Methods

### 2.1. Study Design, Patients, and Data Acquisition

A cross-sectional analytical study was conducted using a dataset from the Colombian Spinal Muscular Atrophy Foundation (FAMECOL), which maintains a comprehensive registry of patients diagnosed with SMA in Colombia from April 2013 to April 2024. Data were provided in tabular format as a .xls file (Excel ver. 2016, Microsoft Corporation, Redmond, WA, USA), including 201 patients diagnosed with SMA by multiplex ligation-dependent probe amplification (MLPA), with cases confirmed by sequencing by a compatible clinical picture and heterozygosity result in MLPA, following the diagnostic algorithm for SMA as published by Mercuri in 2018 [37].

For this study, the variables were grouped into four categories: sociodemographic (age, sex, and origin), clinical (SMA type, tracheostomy, and gastrostomy), and genetic (MLPA results and *SMN2* copy number). Additionally, a grouping by *SMN2* copy number was performed due to the small number of patients, which could result in empty cells when creating contingency tables, limiting the possibility of performing association or impact tests (Group 1—1 and 2 *SMN2* copies, Group 2—3 *SMN2* copies, and Group 3—4 and 5 *SMN2* copies); the groups were determined by the distribution of the number of copies of Angilletta and collaborators in their study [24].

### 2.2. Statistical Analysis

#### 2.2.1. Data Identification, Extraction, and Collection

Data were identified, extracted, and collected from patient records provided by FAMECOL as patients registered with the association, including 201 from April 2013 to April 2024, when the database was delivered. A detailed protocol outlining the inclusion and exclusion criteria was developed to ensure that only patients with confirmed SMA diagnoses and available genetic information on *SMN2* copy numbers were included. Clinical and genetic data were carefully extracted by a team of trained researchers, ensuring accuracy and consistency in data collection. All collected information was anonymised to protect patient identity and stored in a secure, encrypted database.

#### 2.2.2. Dataset Audit

A dataset audit was conducted to ensure the integrity and quality of the collected data. An independent team reviewed the data collection process and data entry into the dataset to identify and correct potential errors or inconsistencies. This process involved cross-checking original records with the information entered into the database, certifying that all entries were accurate and complete. Additionally, periodic audits were conducted to maintain data quality throughout the study, confirming that any updates or corrections were adequately documented.

#### 2.2.3. Formal Statistic Data Analysis

Qualitative variables were presented as absolute and relative frequencies in tables, while quantitative variables were presented as medians with interquartile ranges according to data distribution. Normality was assessed using the Kolmogorov–Smirnov test. Fisher’s exact test was used to analyse the association between SMA types and the number of *SMN2* gene copies, considering that Fisher’s test is useful in cases where the expected frequencies in the contingency table cells are small (less than 5) [38], which can happen in studies of rare diseases like SMA. If there is an imbalance in the distribution of SMA types or the number of SMN2 copies among patients, Fisher’s test can be more accurate than a chi-squared test, which is more sensitive to larger sample sizes and more balanced distributions [38]. Patients were grouped into three subgroups—group 1 (1 and 2 *SMN2* copies), group 2 (3 *SMN2* copies), and group 3 (4 and 5 *SMN2* copies)—groups determined by the distribution of the number of copies of Angilletta and collaborators in their study [24], as can be seen in Table 1. Results were considered statistically significant when the *p*-value was less than 0.05. All analyses were performed using the R statistical programming language (R version 4.4.1, Vienna, Austria). and Stata 16 (College Station, TX, USA: StataCorp LLC) [39,40].

### 2.3. Ethical Considerations

Data collection was conducted under strict confidentiality and ethical protocols. Informed consent was obtained from all participating patients, who signed the corresponding authorisation and habeas data documents. These documents ensured that patients were clearly and comprehensively informed about the study objectives and their rights to privacy and confidentiality of their data. Furthermore, the study adhered to all national and international regulations on protecting personal data and research involving human subjects. Collected data were handled anonymously and coded to prevent direct identification of patients. Only authorised research team members (FAMECOL) had access to the information, and security measures were implemented to protect the data from unauthorised access.

The FAMECOL scientific committee reviewed and approved all study aspects on 24 May 2024, including the research design, data collection and management methods, and measures to protect participants’ rights. This approval ensures that the study was conducted with the highest level of ethical integrity, respecting the dignity and rights of the patients.

## 3. Results

### 3.1. Sociodemographic Characteristics of Participants

A total of 201 patients with a clinical and genetic diagnosis of SMA were included in this study, of which 42% were female (n = 85) and 58% were male (n = 116). The median age of patients at the time of data collection was 9 years, with an interquartile range (IQR) of 4–16 years (Table 1). When stratified by SMA type, the median ages at diagnosis were 2, 7, 14, and 41.5 years for SMA types 1, 2, 3, and 4, respectively.

Regarding the geographical distribution by department, 25.4% of the patients were found in the department of Antioquia (n = 51), followed by Cundinamarca, Risaralda, and Cordoba, each with 5%, as shown in Table 2.

### 3.2. Clinical Features of Participants

It was found that 9% of patients had gastrostomies (n = 18), with 16 being SMA type 1. Tracheostomy management was present in 14 patients (7%). Additionally, 25% (n = 50) were SMA type 1 patients, 36.82% (n = 74) were SMA type 2, 39.35% (n = 59) were SMA type 3, and 8.96% (n = 18) were SMA type 4, as shown in Table 3.

### 3.3. Genetic Features of the Participants

**Figure 1:**
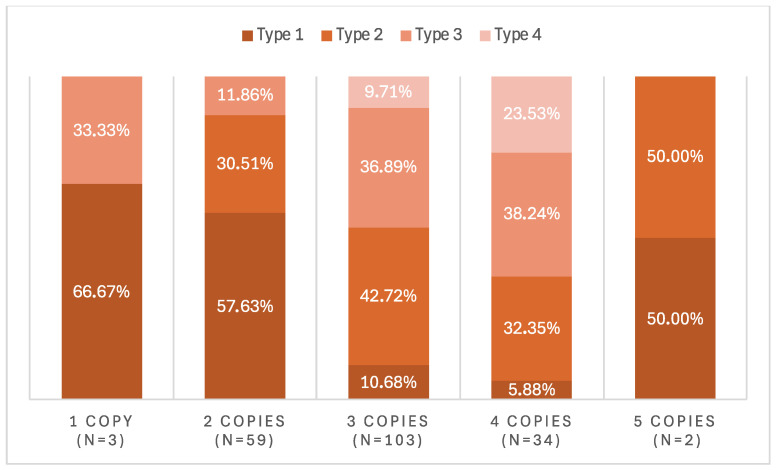
*SMN2* copy number distribution according to AME type.

Molecular analysis of SMA patients revealed that homozygous deletion of exons 7 and 8 was the most frequent genetic alteration (38.8%). However, sequencing was necessary in a small percentage of cases (8.46%) to confirm the diagnosis in patients in whom MLPA resulted in heterozygosity, but clinical and family history gave SMA as the main diagnosis. MLPA proved highly efficient, allowing mutation identification in 94% of patients. Regarding the number of *SMN2* copies, a distribution of 3, 2, and 4 copies was found in 51%, 29%, and 17% of cases, respectively. These results are presented in Table 4 and Figure 1.

### 3.4. SMA Type and SMN2 Copy Number Relationship

Patients were categorised into three groups based on the *SMN2* copy number: group 1 (1 and 2 copies), group 2 (3 copies), and group 3 (4 and 5 copies), as shown in Table 5. Fisher’s exact test to assess the association between SMA type and copy number groups revealed a significant association (*p* < 0.05). This result suggests that the *SMN2* copy number might be consistently related to the SMA type. In particular, it was observed that patients with higher *SMN2* copy numbers tended to present milder forms of the disease, whereas those with fewer copies showed more severe clinical features.

## 4. Discussion

SMA is a neurodegenerative condition caused by *SMN1* gene mutations, resulting in reduced SMN protein expression and subsequent loss of α-motor neurons, leading to severe muscle weakness and often early death. SMA can manifest with a wide range of severity, from lethal infantile forms [41] to milder adult-onset forms; thus, phenotypic variability is a hallmark of this condition [42]. The natural history and clinical findings depend highly on the specific phenotype, leading to a classification system based on the age of onset, severity, and prognosis. Table 1 depicts the main features of SMA types known to date [42,43,44].

Although supportive care remains the cornerstone of SMA management, recent advances in our understanding of the disease have led to innovative therapeutic approaches. This new landscape has underscored the importance of early diagnosis and access to these novel treatments to improve patient outcomes, such as Nusinersen or Risdiplam, already in use in Colombia since 2019 [34] and 2023 [35], respectively, but gene therapy is not yet approved in the country [45]. This study represents a pioneering effort in Colombia, aiming to characterise the SMA population and investigate its epidemiological features, including SMA types and *SMN2* copy numbers. These factors are crucial in optimising diagnosis and treatment strategies in Colombia and Latin America [31,37].

Colombia is committed to the early and timely diagnosis of a wide range of diseases, particularly orphan or rare diseases [33]. This effort aims to reduce the economic costs of medical treatments and mitigate the social, familial, and cultural costs imposed by these conditions. Through implementing public health policies and research programs, the country seeks to improve the quality of life for patients and their families [33,46]. Furthermore, these initiatives are designed to decrease the morbidity and mortality of rare diseases, thereby contributing to a more equitable and sustainable healthcare system [46]. Spinal muscular atrophy falls within this category of pathologies, whose behaviour in Colombia remains poorly understood [29] and for which healthcare centres continue to work towards early and timely diagnosis and patient well-being [47]. This framework is crucial to understanding the social and clinical context related to the challenges of rare diseases. [48]. By considering the broader context, we can develop more effective interventions, implement inclusive health policies, and improve access to care for individuals with rare diseases. This approach ultimately leads to a more equitable and efficient healthcare system [49].

In this context, age at diagnosis remains a critical determinant of clinical outcomes in SMA. Significant delays in suspecting and confirming the diagnosis are frequently observed, hindering access to timely interventions and potentially exacerbating disease progression [50]. Previous studies have reported mean ages of genetic diagnosis of 6.3, 20.7, and 50.3 months for SMA types 1, 2, and 3, respectively [51]. Similarly, a 2023 study conducted in Indonesia found a mean age of diagnosis of 16 months [52]. In contrast, our study revealed a considerably later median age of diagnosis at 9 years for all SMA types, with specific values of 2, 7, 14, and 41.5 years for types 1, 2, 3, and 4, respectively. These findings align with those reported in the Brazilian population [53]; other countries in the region, such as Argentina, show different ages at diagnosis of patients, as evidenced in the FAME registry published in 2023 by Vazquez et al., where a diagnostic age for SMA 1 of 5.2 months (IQR 3.37–7.43), for SMA 2 of 18.68 months (IQR: 14.18–27.77), for SMA 3 of 49.87 months (IQR 29–118) and for SMA 4 of 28 years (IQR 26–34) was evident, figures that are far from the results of the present study [54]. The observed delay in diagnosis highlights a critical gap in the current diagnostic pathway. It underscores the need for improved awareness and earlier intervention, a critical point that has changed in European countries [55,56] and even in the USA [57], where newborn screening for SMA is widely used and where even patients can be treated presymptomatically, which could also be considered as an option in the Colombian population [58].

Concerning the distribution by sex, the results of this study are consistent with those reported by Souza et al., who found that 55% of adult patients diagnosed with type 4 SMA were male [53]. Similarly, Ar Rochmah et al. reported that 50.81% of their sample also consisted of males [59], which is consistent with our observations, as well as Jianli Sun et al. in their analysis of the dataset, where the male/female ratio correlates with the incidence and prevalence of SMA in various countries [60]. However, it is important to highlight that, despite the extensive literature suggesting there are no significant differences in disease presentation between men and women, such as the studies above and the results of this one, it has been observed that in families with a diagnosis of SMA, a greater number of male members tend to be affected [60]. Also, some studies suggest the possibility of greater genetic susceptibility in males compared to females in SMA [59,61], which could explain this greater frequency in the presentation of the disease in men than in women, although it is still debated.

Regarding the location of the patients, it is observed that the highest number of cases are concentrated in areas such as Antioquia, Cundinamarca, and some from the Caribbean region. This trend may be related to the frequency of endogamy and consanguinity in these areas, as suggested by studies of isonymy and the local behaviour of the population [62,63]. It is important to highlight that although municipalities like Boyacá have documented a 15% rate of endogamy [64], they do not show a significant number of patients with SMA, unlike other diseases with an identified genetic component, such as mucopolysaccharidosis, Alzheimer’s disease, type I bipolar affective disorder, juvenile Parkinson’s disease, and congenital anomalies, some of them even autosomal recessive such as SMA [65]. This fact may suggest underdiagnosis or under-reporting of the disease in certain regions [66]. These findings can be associated with the Colombian genetic variability due to the multiple migrations and colonising processes, as evidenced in the study by Chande et al., who mention that Colombia has a diverse and multiethnic population with important ancestral contributions from Europe, Africa, and the Americas [67], supported by the study of Luis G. Carvajal-Carmona in the Antioquia population [68], and by Rishishwar et al., who referred to this massive transfer of life in Colombia [69].

Gastrostomy and tracheostomy are some of the invasive interventions for managing patients with more aggressive disease and SMA types 1 and 2, to increase patient survival. As mentioned in the study by Lemoiney et al., Kaplan–Meier curves demonstrated that children who received early proactive respiratory support had statistically longer survival compared to those who received supportive care [70]; in this case, it was found that 8.96% of patients had tracheostomies, 16 of whom had type 1 SMA. Gastrostomy management was present in 14 patients (6.96%). Recently, it has been proposed to increase proactive non-invasive management to reduce comorbidities and complications in patients with SMA types 1 and 2 [71]. It is worth mentioning that although these two options are invasive in the management of patients, they have proven in some cases to be necessary to prolong their lives, but it is necessary to add that a timely diagnosis and early therapeutic management of patients can make a complete difference [58,72,73,74].

It is also important to assess not only the patients’ clinical characteristics but also the population’s genetic characteristics, including the diagnostic test used. A meta-analysis of 33 studies published in 2020 [75] with 3393 SMA patients studying the copy number quantification techniques for SMN2 revealed that TaqMan (TaqMan^®^ Real-Time PCR Assays. Waltham, MA, USA: Thermo Fisher Scientific), LightCycler (*LightCycler^®^ 96 System*. Basel, Switzerland: Roche Diagnostics), MLPA, PCR-CE, and digital PCR were widely used. This study evidenced that 54% of patients (n = 1870) were evaluated using MLPA, LightCycler was used in 21.4% of cases (n = 741), and TaqMan in 6.5% (n = 28) [75]. In the present study, 100% of patients were analysed by MLPA, which has a diagnostic sensitivity and specificity of 95% and 99%, respectively [76], although some required subsequent sequencing, achieving not only diagnosis but also identification of the *SMN2* copy number. MLPA has limitations like any test, as Alicia Gomes MS et al. mention in their 2018 book, where they say that MLPA only detects copy number variations but cannot identify the exact breakpoints of a mutation [77]. Although it indicates the presence of a deletion or duplication, it does not provide details about where the alteration begins or ends [78]. If the breakpoints of a variant are located between two probes in the assay, MLPA cannot precisely define its location within that region. In addition, its scope is limited to specific genes or regions. For example, if the analysis shows duplication in all probes, it is impossible to determine whether the cause is a chromosomal abnormality or a duplication involving multiple genes [77]. Additional techniques are needed to obtain these details. In addition, MLPA has a reduced capacity to detect mosaic deletions or duplications when the variant affects less than 30% of the cells analysed [77].

Each of these techniques has advantages and disadvantages, in addition to other technical factors to consider, such as DNA quality and interpretation and control [78]. Digital PCR methodologies and new protocols based on next-generation sequencing (NGS) can be especially effective for resolving complex cases [78]. NGS technology allows for a comprehensive analysis of *SMN2* copies at the genomic level, including introns, and facilitates a more accurate assessment of the equivalence and quality of *SMN2* copies. Additionally, NGS provides valuable data that can be validated to establish more complete correlations between genotype and phenotype [79]. In the present study, NGS was used in 8.46% of cases, as the previous MLPA result had been simple heterozygosity, but the patient’s clinical presentation remained suggestive of SMA.

Accurate estimation of *SMN2* copy number is crucial for establishing precise genotype–phenotype correlations, predicting disease progression, stratifying patients, and identifying those eligible for specific treatments [75]. However, in some patients, this information may not be sufficient to correlate with the observed phenotype. To date, the number of copies of the *SMN2* gene and the presence of rare *SMN2* variants (such as NM_017411.3:c.859G>C and NM_017411.3:c.835–44A>G) remain the primary modifiers of the disease phenotype. In the present study, a statistically significant difference was observed in the association between the number of *SMN2* copies and the type of SMA, consistent with findings from Spanish studies in 2018 [80] and Argentine studies in 2016 [81]. Despite this, there is research that contradicts these findings, such as a systematic review by Powis et al. in 2023, which mentions that while many articles claim that an increase in the number of *SMN2* copies leads to a milder SMA phenotype [82], the reported cases do not fully support this concept, adding that there seems to be a lack of correlation between a mild phenotype and *SMN2* copy number ≥ 4, a fact also demonstrated by intrafamilial variability [82], which could explain the finding in this study of three patients where, despite having more than 3 copies in the *SMN2* gene, the patients had an aggressive type of SMA (SMA type 1).

While infrequent, discrepancies between copy number and disease progression can be observed in clinical practice, as seen in this study where two patients had 5 copies of *SMN2* but presented with the clinical phenotype of type 1 and type 2 SMA, as also reported in the literature for US and Spanish populations [80,81,83]. Conversely, cases where patients with 1 copy of *SMN2* present with a type 4 (mild) phenotype can also be observed, as with one of the patients in the present study where approximately eight patients presented with 1 and 2 copies but had type 3 SMA, also related to the literature from studies by Tizzano and colleagues [36,75]. Several studies emphasise the association between the number of *SMN2* copies and the type and severity of SMA [13,80,81] (Table 6), but they also mention that other factors could determine the variability shown in their results [13], such as the 2020 Jones et al. study between siblings with different SMA phenotypes [84]. Recently, in addition to the *NAIP* gene [85,86], other factors have been reported, such as plastin 3 as a protective factor for axonal growth [87] and neurocalcin D, which restores endocytosis at the neuromuscular junction [88], which is why it is becoming increasingly important to evaluate other factors that allow for determining the prognosis of patients with SMA.

These uncertainties and the presence of type 1 individuals with ≥4 copies of *SMN2* led to a review of the guidelines proposed by Glascock et al., who provide recommendations for immediate treatment for paediatric patients diagnosed with 4 copies of *SMN2* and “watchful waiting” for individuals with 5 copies of *SMN2* [99], regarding management and treatment.

Despite this, there is still no evidence that the genes most commonly identified (*NAIP*, *p44*, and *H4F5*) are involved in modifying the phenotypes of patients with SMA [86]. Deletions of these genes in individuals with type 1 SMA likely reflect large-scale deletions involving the *SMN1* gene and some copies of the *SMN2* gene, leading to a severe phenotype due to a lack of the SMN protein product [86]. Although identifying these modifiers was not the primary objective of this study, they should be considered as an option for evaluating disease progression in the future.

This pioneering study in Colombia has enabled a deeper understanding of the population of patients with SMA, providing valuable data on the disease’s distribution, behaviour, and sociodemographic and genetic characteristics. Although the number of copies of the SMN2 gene remains an important indicator, our research and the scientific literature suggest that the quality of these copies and other genetic factors may play a crucial role in disease severity. These discrepancies underscore the need for a comprehensive evaluation that focuses not only on the quantity of *SMN2* copies but also on their quality and identifying other potential genetic modifiers. In this way, we can develop more effective and personalised strategies for managing and treating SMA, significantly improving the quality of life for patients.

One of the main limitations of this study is the number of patients included, which could affect the generalisability of the results. The scarcity of studies with large samples limits the ability to perform more robust analyses and detect subtle differences between subgroups. Furthermore, the absence of detailed clinical data, such as age of symptom onset, functional scale scores, and family history, makes it difficult to comprehensively assess the relationships between variables to understand the patient’s clinical context better. This lack of information could lead to biased interpretations and a limited understanding of the disease in the population studied. Future research should aim to include a larger number of participants and collect more clinical data to address these limitations. Alongside this, confounding variables, such as environmental factors, socioeconomic status, or differences in healthcare access, could influence disease progression or diagnosis. It is also relevant to consider the memory bias that patients may present, especially those diagnosed before the creation of FAMECOL and the patients’ affiliation with it.

## 5. Conclusions

This study represents one of the largest characterisations of SMA patients in Colombia and highlights the urgent need for earlier diagnosis and personalised treatment strategies based on *SMN2* copy number.

Our findings underscore Colombia’s expedited diagnostic process for SMA. Despite a significantly younger age at genetic diagnosis in other countries, the substantial delay in diagnosis in our cohort not only hinders timely access to disease-modifying therapies but also exacerbates motor neuron apoptosis. These findings emphasise the importance of establishing early detection protocols and comprehensive clinical follow-up.

Furthermore, our study revealed a unique geographic distribution of SMA patients in Colombia, possibly linked to endogamy and consanguinity. These findings suggest that Colombia’s genetic diversity and consanguinity patterns may influence the prevalence and expression of rare genetic disorders like SMA. This factor highlights the importance of considering these factors in designing public health programs and genetic intervention strategies, such as newborn screening, which have been shown to have important value in the timely diagnosis and treatment of patients.

In conclusion, while the number of *SMN2* gene copies significantly determines SMA severity, our research indicates that the quality of these copies and other genetic factors play a crucial role, emphasising the need for a comprehensive assessment considering both the quantity and quality of *SMN2* copies and the identification of other potential genetic modifiers for improved disease prediction and management.

## Figures and Tables

**Table 1:** Classification of SMA.

SMA Type	Eponymous	Median Survival	Motor Function	*SMN2* Copy Number
Type 0	Congenital SMA	4 weeks	None reached	0
Type 1	Werdnig–Hoffman Diseses	<2 years	Some head control	1–2
Type 2	Dubowitz Diseases	>25 years	Sit	2–3
Type 3	Kugelberg–Welander Diseases	Adult	Stand or ambulatory	3–4
Type 4	Adult SMA	Adult	All motor function	≥4

Adapted from the original by Arnold et al. [23] and Angilletta et al. [24].

**Table 2:** Sociodemographic features of the participants.

Variables	n	%
Sex		
Male	116	58
Female	85	42
Department		
Antioquia	51	25
Arauca	1	0.50
Atlántico	10	5
Bolívar	9	4
Boyacá	4	2
Caldas	4	2
Casanare	1	0.50
Cauca	4	2
Cesar	1	0.50
Cundinamarca	39	19
Córdoba	10	5
Huila	5	2
La Guajira	3	1
Magdalena	1	0.50
Meta	4	2
Norte de Santander	7	3
Quindío	8	4
Risaralda	10	5
Santander	3	1
Sucre	6	3
Tolima	2	1
Valle del Cauca	13	6

**Table 3:** Clinical characteristics of the participants.

Variable	n	%
Gastrostomy	19	9
Tracheostomy	14	6
SMA type		
Type 1	50	25
Type 2	74	37
Type 3	59	29
Type 4	18	9

**Table 4:** Genetic features of the participants.

Variable	n	%
Type of diagnostic test		
MLPA	188	94
Sequencing	13	6
Genetic study result		
Homozygosity (2 copies)	184	92
Compound heterozygote	17	8
Mutation type		
Exon 7 to 8 deletion	78	38.8
Exon 7 deletion	102	50.7
Exon 7 to 8 duplication	1	0.5
Point mutation	20	10
*SMN2* copies		
1 copy	3	2
2 copies	59	29
3 copies	103	51
4 copies	34	17
5 copies	2	1

**Table 5:** SMA types according to SMN2 copy number.

SMA Type	*SMN2* (Copy Number)		
	Group 1 (1–2 Copies)	Group 2 (3 Copies)	Group 3 (4–5 Copies)
Type 1	36	11	3
Type 2	18	44	12
Type 3	8	38	13
Type 4	0	10	8

**Table 6:** Clinical epidemiological studies that evaluated the correlation or association between the number of SMN2 copies and the type of SMA.

Article	Correspondence Author	Year	Country	n	Type of Study
1	Calucho et. al [80]	2018	Spain	625	Correlation
2	Ricci et al. [13]	2023	Italy	169	Association
3	Medrano et. al. [81]	2016	Argentina	144	Correlation
4	Mailman et al. [20]	2002	USA	610	Correlation
5	Zarkova et al. [89]	2015	Serbia	44	Association
6	Kesari et al. [90]	2005	India	50	Correlation
7	Petite et al. [91]	2011	France	103	Correlation
8	Jedrzejowska et al. [92]	2009	Poland	1039	Association
9	Arkblad et al. [93]	2009	Sweden	47	Correlation
10	Amara et al. [94]	2012	Tunisia	26	Correlation
11	Wirth et al. [95]	2006	Germany	115	Correlation
12	Zhang et al. [96]	2020	China	40	Association
13	Harada et al. [97]	2002	Japan	27	Correlation
14	Elsheikh et al. [98]	2009	USA	45	Association

## Data Availability

These data are available as full content in Harvard Dataverse. Lamadrid, Jose, 2024, “Spinal Muscular Atrophy in Colombia: A dataset in 201 patients”, https://doi.org/10.7910/DVN/6H2CEZ (accessed on 16 October 2024), Harvard Dataverse, V1.
