# Correction: Lamadrid-González et al. *SMN2* Copy Number Association with Spinal Muscular Atrophy Severity: Insights from Colombian Patients. *J. Clin. Med.* 2024, *13*, 6402

**DOI:** 10.3390/jcm15135062

**Published:** 2026-06-29

**Authors:** José Lamadrid-González, Sandra Castellar-Leones, Julio César Contreras-Velásquez, Valmore Bermúdez

**Affiliations:** 1 Programa de Maestría en Genética, Centro de Investigaciones en Ciencias de la Vida, Universidad Simón Bolívar, Barranquilla, Atlántico 080003, Colombia; 2 Departamento de Medicina Fisica y Rehabilitacion, Facultad de medicina, Universidad Nacional de Colombia, Bogotá DC 111321, Colombia; smcastellarl@unal.edu.co; 3 Departamento de Productividad e Innovación, Universidad de la Costa, Barranquilla, Atlántico 080002, Colombia; 4 Facultad de Ciencias de la Salud, Centro de Investigaciones en Ciencias de la Vida, Universidad Simón Bolívar, Barranquilla, Atlántico 080003, Colombia

## Back Matter Correction

There was an error in the original publication [1] in the “Conflicts of Interest: The authors declare no conflicts of interest”. The authors requested to update the Conflicts of Interest as below.

**Conflicts of Interest:** José Lamadrid-González: an employee of BIIB Colombia SAS, a local subsidiary, belonging to Biogen. Sandra Castellar-Leones: received honoraria for speaking at symposia from Roche, Novartis and participated on Biogen’s advisory board, the latter being a subsidiary of Biogen. The rest authors have no conflicts to declare.

The authors state that the scientific conclusions are unaffected. This correction was approved by the Academic Editor. The original publication has also been updated.

## References

[1] Lamadrid-González J., Castellar-Leones S., Contreras-Velásquez J.C., Bermúdez V. (2024). *SMN2* Copy Number Association with Spinal Muscular Atrophy Severity: Insights from Colombian Patients. J. Clin. Med..

